# Effects of Essential Oil from Hinoki Cypress, *Chamaecyparis obtusa*, on Physiology and Behavior of Flies

**DOI:** 10.1371/journal.pone.0143450

**Published:** 2015-12-01

**Authors:** Shin-Hae Lee, Hyung-Seok Do, Kyung-Jin Min

**Affiliations:** Department of Biological Sciences, Inha University, Incheon, Korea; Tohoku University, JAPAN

## Abstract

Phytoncides, which are volatile substances emitted from plants for protection against plant pathogens and insects, are known to have insecticidal, antimicrobial, and antifungal activities. In contrast to their negative effects on microorganisms and insects, phytoncides have been shown to have beneficial effects on human health. Essential oil from Hinoki cypress (*Chamaecyparis obtusa*) is mostly used in commercial products such as air purifiers. However, the physiological/behavioral impact of essential oil from *C*. *obtusa* on insects is not established. In this study, we tested the effects of essential oil extracted from *C*. *obtusa* on the physiologies and behaviors of *Drosophila melanogaster* and *Musca domestica*. Exposure to essential oil from *C*. *obtusa* decreased the lifespan, fecundity, locomotive activity, and developmental success rate of *D*. *melanogaster*. In addition, both fruit flies and house flies showed strong repellent behavioral responses to the essential oil, with duration times of about 5 hours at 70 μg/ml. These results suggest that essential oil from *C*. *obtusa* can be used as a ‘human-friendly’ alternative insect repellent.

## Introduction

In nature, plants are usually exposed to various environmental stresses such as dehydration, pollutants, UV radiation, pathogen infection, and attack by herbivorous insects. In response to these stresses, plants have developed their own defense systems [1]. Phytoncides are volatile organic compounds emitted by plants that protect against plant pathogens and insects [2]. Phytoncides possess strong antimicrobial and insecticidal activities and are applied in many fields as food preservatives and insect repellents [3–5]. For example, essential oils from herbs such as rosemary, oregano, clove, and thyme are reported to have antimicrobial activities against *Listeria*, *Salmonela*, and *E*. *coli* O157 [5]. In addition, essential oil from *Artemisia absinthium* is toxic to developing larvae of *D*. *melanogaster* [6].

In contrast to their negative effects on microorganisms and insects, phytoncides have been shown to have beneficial effects on human health. For example, cellular damage by reactive oxygen species and UV-induced matrix metalloproteinase-1 activity was shown to be significantly reduced by phytoncides in human dermal cells [7]. Phytoncides extracted from a mixed homogenate of 118 plants also was found to reduce the level of noradrenaline, a stress hormone, in stroke-prone spontaneously hypertensive rats [8]. In addition, in East Asian countries, people are commonly fascinated by walking among forests, a practice known as “forest bathing”, to improve their health [9, 10]. The number of natural killer cells and levels of intracellular anticancer proteins were reported to increase when subjects were exposed to phytoncides during forest bathing [11]. In this regard, many related commercial products such as air purifiers or deodorants have been developed.

Hinoki cypress, *Chamaecyparis obtusa*, is the representative tree of forest bathing, and essential oil from *C*. *obtusa* is widely used in commercial products. This essential oil promotes proliferation and division of hair follicle cells through induction of vascular endothelial growth factor [12, 13] and has anti-atopic activity in mice [14]. Furthermore, β-thujaplicin (hinokiol), one of the constituents of *C*. *obtusa* essential oil, was recently reported to suppress proliferation of breast cancer cells [15] and have anti-inflammatory effects in mice [16].

Although essential oil from *C*. *obtusa* is widely used and its beneficial effects are well investigated, the physiological/behavioral impact of this essential oil on insects is not well established. Park et al (2003) previously showed that some components of this essential oil were able to induce over 90% adult mortality in *Callosobruchus chinensis* within 1–2 days [17], although the long-term effects of essential oil on insect physiology and behavior are not well known. Moreover, the concentrations of essential oil used previously do not reflect the actual concentrations used in commercial products. In this study, we tested the effects of essential oil extracted from *C*. *obtusa* on the physiologies and behaviors of fruit flies (*D*. *melanogaster*) and house flies (*Musca domestica*). Effects of essential oil on development, longevity, fecundity, and locomotive activity were measured using fruit flies, and repellent activity was measured using fruit flies and house flies.

## Materials and Methods

### Fly strain and maintenance

A study was conducted using Canton-S, a wild-type strain of fruit fly (*D*. *melanogaster*), which was provided from the Bloomington stock center (Indiana University, USA). Larvae of house flies, *M*. *domestica*, were purchased from a local fishing gear shop (Cheongyang gear shop, Cheongyangri-dong, Seoul, Korea) and reared into adults in the laboratory. Both flies were maintained in the culture room at 24°C with 45% humidity and exposed to light and dark for 12 hours. Standard cornmeal-sugar-yeast with agar (CSY) medium [18] was used for rearing larvae of fruit flies and house flies. When flies were eclosed as adults, standard sugar-yeast (SY) medium [18] was provided to fruit flies, whereas sugar and water were provided to house flies [19]. Flies exposed to essential oil from *C*. *obtusa* were reared in a separate incubator to prevent the effects of phytoncides on non-exposed flies by diffusion.

### Exposure to *C*. *obtusa* essential oil

Steam distillated essential oil extracted from leaves and branches of *C*. *obtusa* was purchased from “In The Forest Co., Ltd.” (Seoul, Korea) and delivered to flies at concentrations of 25 or 70 μg/ml after dilution with distilled water. Final concentrations of the essential oil were determined based on the actual concentrations used in commercial products. Essential oil from *C*. *obtusa* was delivered by feeding or fumigation. Detailed method of delivery is described below.

### Lifespan assays

Newly eclosed 100 male and 100 female adult fruit flies were transferred to 500 cm^3^ demography cages, and three replicate cages were set up for each group. For essential oil delivery by fumigation, two vials were affixed into a demography cage—one for food delivery and one for essential oil fumigant delivery. A vial containing normal fresh media was affixed to one side, and a vial containing filter paper soaked with 100 μl of diluted essential oil was affixed to another side. Mesh was placed inside of the vial containing essential oil to block direct contact with flies. For delivery of essential oil by feeding, undiluted essential oil was mixed with food media with a final concentration of 25 or 70 μg/ml, and this food vial was affixed into the demography cage. The vials containing fresh SY media and filter paper soaked with essential oil were changed every 2 days, at which time the number of dead flies was counted.

### Pupation frequency

Canton-S female flies were maintained on a 90 mm plate containing CSY food for 12 hours for oviposition, and laid eggs were transferred into polystyrene vials with a fine brush at a density of 10 eggs per vial. Twenty replicate vials were set up for each group. For delivery of essential oil by fumigation, a cotton vial plug was soaked with an appropriate 100 μl of essential oil. For delivery of essential oil by feeding, the eggs were transferred to and reared on essential oil-mixed food media. The number of newly formed pupae was checked daily. Pupation frequency was given by the total number of pupae divided by the number of eggs laid.

### Locomotion activity

Climbing ability of flies was measured by using rapid iterative negative geotaxis (RING) assay [20] with some modifications. Adult fruit flies were exposed to essential oil fumigant for 10 days via soaking cotton vials with 100 μl of diluted essential oil or distilled water and then transferred to glass vials—10 males and 10 females in each vial. These vials were loaded into the apparatus and tapped on a table three times in rapid succession to initiate a negative geotaxis response. The positions of flies were captured by a digital camera 4 sec after initiation of behavior, and the number of flies that climbed above the standards (40 mm and 80 mm from the bottom) was counted. After each trial, the flies were allowed 1 min of recovery from shock. These cycles were conducted four times with 10 replicates in each group.

### Fecundity assay

Within the first 24 hours of eclosion, adult fruit flies were collected, and each vial containing fresh SY media was set up with two males and one female. A cotton vial plug was soaked with 100 μl of essential oil. Flies were transferred every 24 hours to new vials with fresh cotton plugs soaked with essential oil or distilled water, and the number of laid eggs was counted for 5 days [21].

### Repellent test

To test the repellent activity of essential oil of *C*. *obtusa*, we used T-maze assay with minor modifications [22]. Briefly, two food vials—one with and one without essential oil—were installed at opposite sides of the cage described in the lifespan assay. A funnel made of filter paper was inserted into each vial so that flies could move in only one direction. Cotton plugs soaked with 100 μl of essential oil or distilled water were placed at the bottom of the vials, and 100 male flies were transferred to the cage after starvation for 4 hours in advance. The number of flies moved into each vial was counted every 10 minutes for at least 5 hours. Three replicates were established for each group.

### Measurement of phytoncides duration time

Duration time of repellency was measured by a similar method as the repellent test. Demography cages were set up with two vials (with essential oil or distilled water), and 100 μl of essential oil was supplemented to the cotton plugs, which were placed at the bottom of food vial. Flies were transferred to the cage immediately or 2, 4, or 6 hours after administration of essential oil, and the numbers of flies that moved to each vial were counted every 10 minutes. Three replicates were established for each group.

### Statistical analysis

Standard survival models in the JMP software (SAS Institute, Cary, NC, USA) were used for log-rank test of survivorship. One-way analyses of variance (ANOVA) or Chi-squared test were performed to compare the data in the development, fecundity, locomotion tests, and repellent test. Asterisks on figures were used to indicate significant differences compared to the control.

### Gas chromatography-mass spectrometry

The information on chemical composition of *C*. *obtusa* essential oil was provided by the manufacturer (In The Forest Co. Ltd.). Essential oil from *C*. *obtusa* leaves and branches was analyzed using a gas chromatograph-mass selective detector (Agllent 7890A). The gas chromatography conditions were as follows: GC column, HP-5MS; injector temperature, 270°C; carrier gas, Helium; rate, 1 mL/min; oven temperature, 40°C to 250°C at 3°C/min.

## Results

### Effects of *C*. *obtusa* essential oil on survival of *D*. *melanogaster*

Essential oils usually contain 20–60 components. Gas chromatography mass spectrometry analysis (GC/MS) revealed that the essential oil from *C*. *obtusa* used in our experiments contains several terpenes. The main components were identified as α-Terpinolene (19.45%), (+)-3-Carene (15.17%), α-Pinene (10.12%), Sabinene (6.27%), and γ-Terpinene (4.77%) (Table 1).

**Table 1:** Major components in essential oil from *C*. *obtusa* identified by GC-MS analysis.

Number	Compound	RT (min)^a^	Area (%)^b^	Quality (%)
1	α-Pinene	9.07	10.12	97
2	Camphene	9.62	1.89	98
3	Sabinene	10.69	6.27	96
4	2-β-Pinene	10.78	2.20	97
5	β-Mycene	11.48	1.18	96
6	(+)-3-Carene	12.28	15.17	97
7	α-Terpinene	12.55	2.85	98
8	ο-Cymene	12.90	1.82	97
9	γ-Terpinene	14.47	4.77	97
10	α-Terpinolene	15.87	19.45	98
11	L-Bonyl acetate	24.90	2.07	99

^a^ Retention time.

^b^ The relative amount of the sub-fraction.

**Fig 1:**
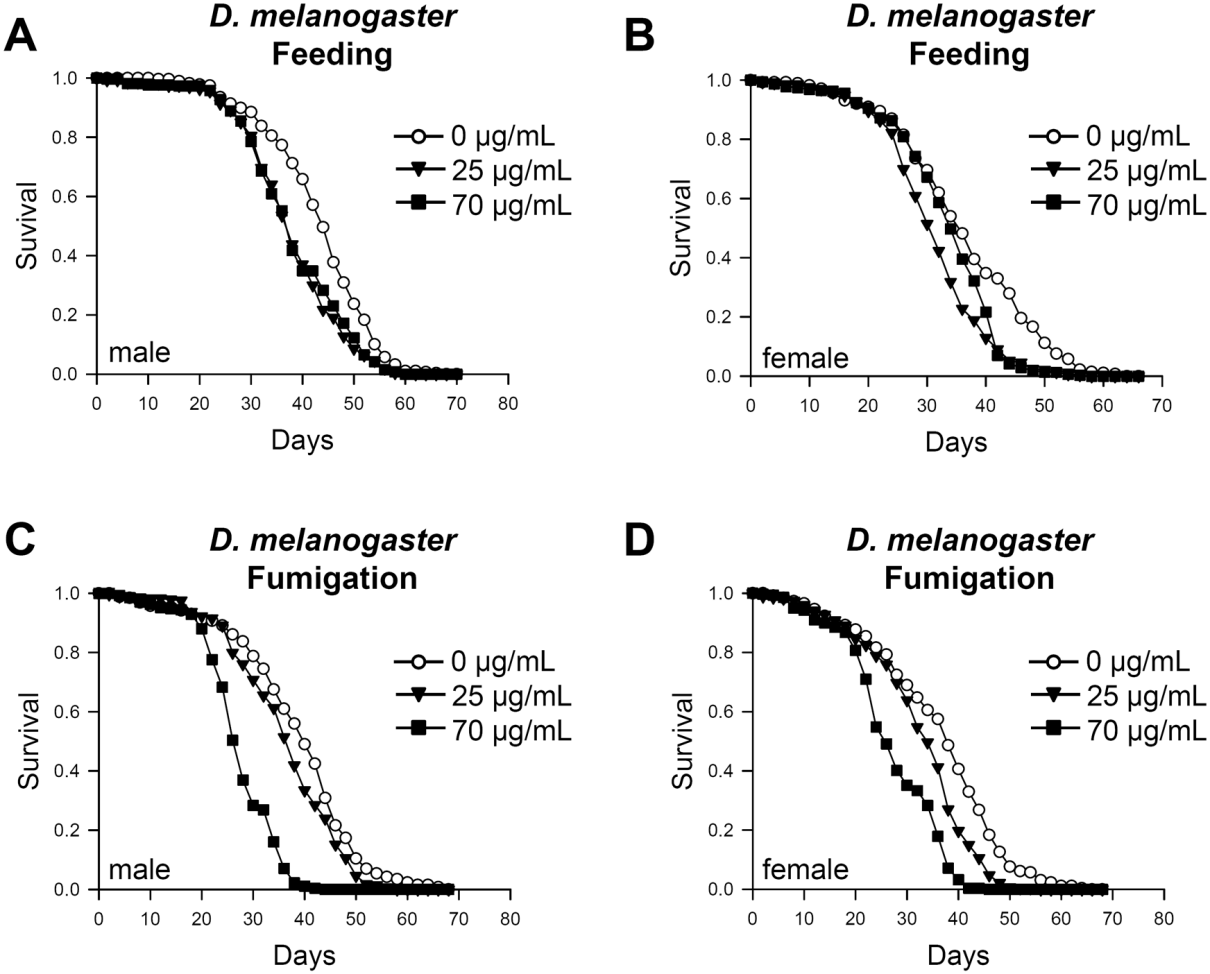
Essential oil from *C*. *obtusa* reduces adult lifespan of fruit flies. Lifespan of male and female flies decreased upon exposure to essential oil from *C*. *obtusa* by feeding (A, B) or fumigation (C, D). (A, C) Male. (B, D) Female.

The survival rate of fruit flies exposed to essential oil from *C*. *obtusa* was measured. The essential oil was delivered either by food ingestion or fumigation. Ingestion of essential oil with a food source significantly reduced the survival rates of flies in both sexes compared to the untreated group (Fig 1A and 1B, p <0.0001). Flies exposed to *C*. *obtusa* fumigant also showed significantly reduced survival rates in both sexes in a dose-dependent manner (Fig 1C and 1D, p <0.0001). The mean lifespan of flies exposed to fumigant of the essential oil (70 μg/ml) was significantly reduced by 29.5% in males and 25.2% in females (Table 2). Our data show that essential oil from *C*. *obtusa* is toxic to *Drosophila melanogaster*, and the effect is greater when delivered via fumigation versus food ingestion. Therefore, essential oil was delivered by fumigation in most of the following experiments.

**Table 2:** Lifespan of fruit flies exposed to *C*. *obtusa* essential oil.

Exposure		Sex	Number of Flies	Mean Lifespan (day)	Maximum Lifespan (day)	P-value
Feeding	0 μg/mL	male	279	43.48±0.6	68	
	25 μg/mL		267	37.9±0.6	60	<0.0001
	70 μg/mL		261	38.25±0.64	60	<0.0001
	0 μg/mL	female	276	36.61±0.71	64	
	25 μg/mL		286	31.36±0.53	58	<0.0001
	70 μg/mL		314	33.56±0.52	58	<0.0001
Fumigation	0 μg/mL	male	259	38.9±0.74	68	
	25 μg/mL		284	36.43±0.6	58	<0.0001
	70 μg/mL		268	27.43±0.43	42	<0.0001
	0 μg/mL	female	262	36.47±0.77	66	
	25 μg/mL		274	32.75±0.63	56	<0.0001
	70 μg/mL		279	27.29±0.54	46	<0.0001

### Effects of *C*. *obtusa* essential oil on development

**Fig 2:**
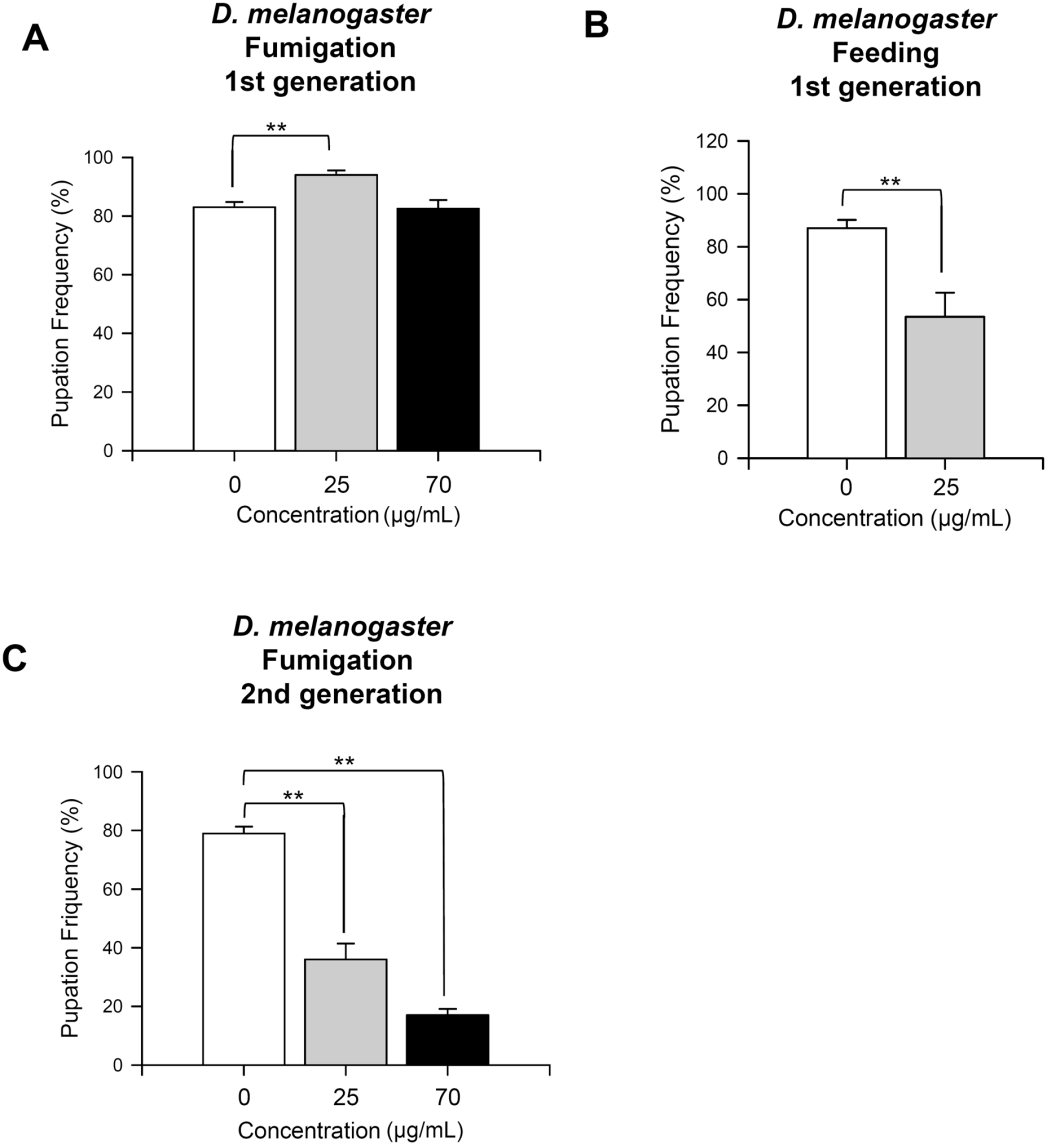
Fumigation or feeding of *C*. *obtusa* essential oil affects pupation ratio of fruit flies. (A) Transformation rate of eggs to pupae (pupation frequency) was not significantly altered by exposure to 70 μg/ml of *C*. *obtusa* essential oil fumigant, whereas fumigation of 25 μg/ml of essential oil slightly but significantly increased pupation frequency. (B) Pupation frequency of fruit flies was significantly reduced upon feeding of food mixed with essential oil. (C) Pupation frequency of offspring was significantly reduced upon exposure of parents to *C*. *obtusa* essential oil fumigant. **p<0.001.

Volatile chemicals emitted by plant oils such as cardamom oil as well as essential oils from poaceae and eucalyptus have been reported to prevent development of insects [23–25]. Effect of fumigant of *C*. *obtusa* essential oil on development of fruit flies was checked by measuring pupation ratio, which is the percentage of successful transformation from larvae to pupae, after exposure to essential oil fumigant. Unexpectedly, no significant difference in pupation ratio was detected in the 70 μg/ml of essential oil-treated group, whereas the ratio was slightly but significantly elevated in the 25 μg/ml of essential oil-treated group (Fig 2A, ANOVA test, p <0.001). Furthermore, the pupation ratio was significantly reduced by ingestion of food containing 25 μg/ml of essential oil (Fig 2B, ANOVA test, p<0.001). Since larvae of *D*. *melanogaster* cultured in the laboratory spend most of their lives feeding inside of semi-solid food, fumigants of the essential oil may not significantly affect the development of larvae residing inside food. In a subsequent experiment, parental flies were exposed to fumigants of essential oil for 10 days, and pupation ratio of offspring was examined. Pupation ratio markedly decreased in offspring of essential oil-exposed parents compared to the control (Fig 2C, ANOVA test, p <0.001).

### Effect of *C*. *obtusa* essential oil on locomotive activity

**Fig 3:**
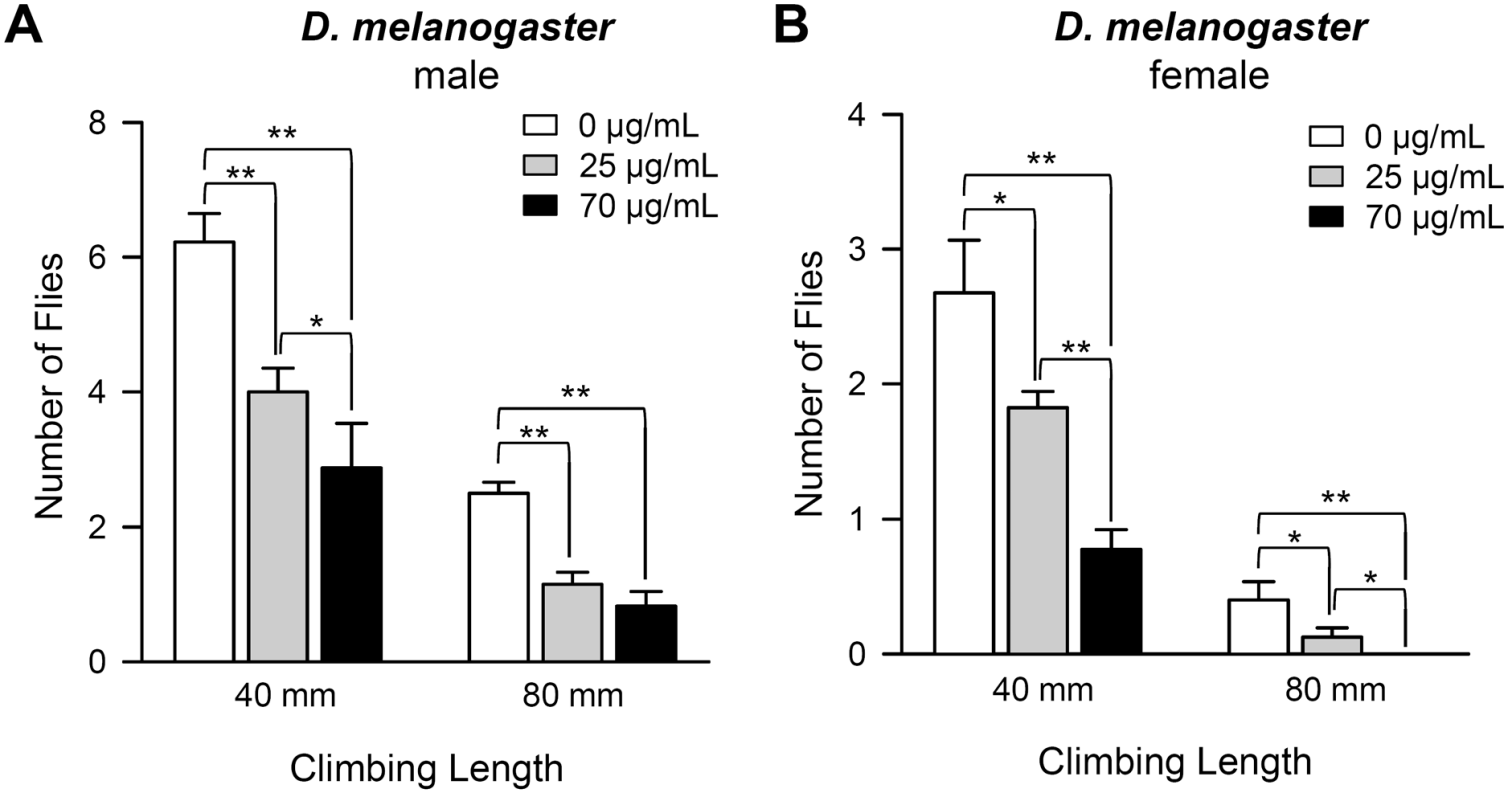
Fumigation of *C*. *obtusa* essential oil reduces locomotive activity of fruit flies. Climbing ability was reduced by fumigation of *C*. *obtusa* essential oil. (A) Male. (B) Female. *p<0.05, **p<0.01.

We next examined the motility of flies in response to *C*. *obtusa* essential oil. After exposure of flies to fumigants of essential oil for 10 days, negative geotaxis behavior of flies was observed. The climbing ability of flies exposed to fumigants of essential oil was significantly reduced in both males (Fig 3A, ANOVA test, p <0.001) and females (Fig 3B, ANOVA test, p <0.05) compared to the control. Especially, no female flies climbed up to 80 mm on test tubes when exposed to 70 μg/ml of essential oil fumigant.

### Effect of *C*. *obtusa* essential oil on fecundity

**Fig 4:**
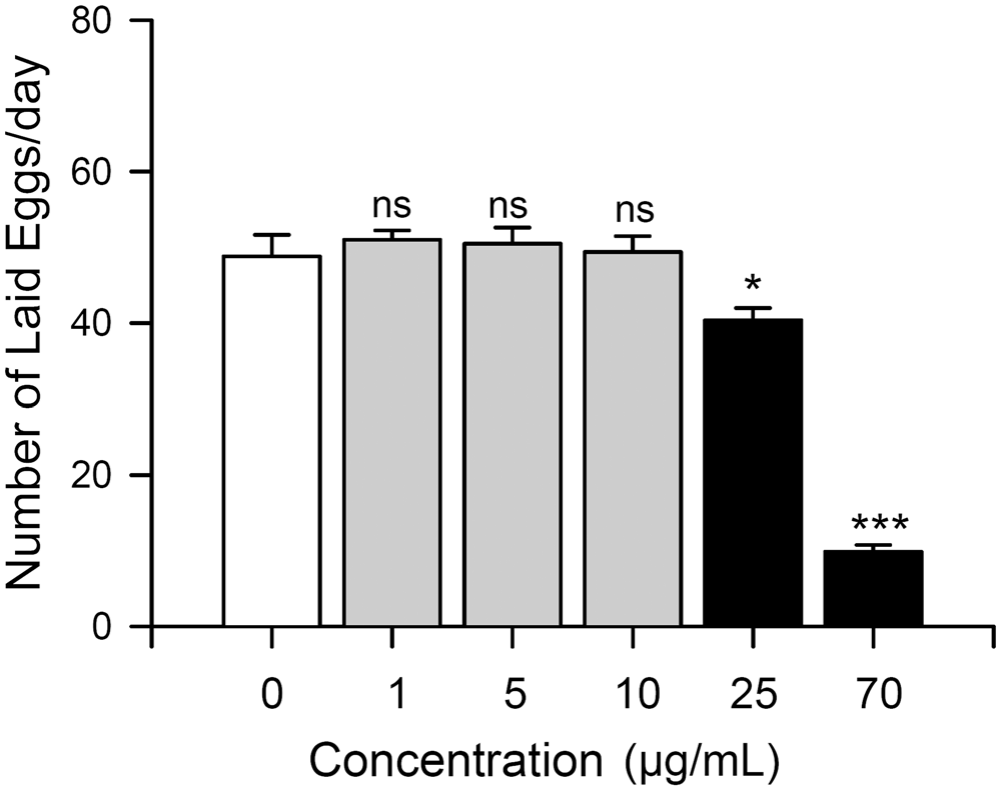
Fumigation of *C*. *obtusa* reduces fecundity of female fruit flies. Average number of eggs laid per day by female fruit flies significantly decreased upon exposure to 25 or 70 μg/ml of *C*. *obtusa* essential oil fumigant (black bars) but was not affected by exposure to 1–10 μg/ml of *C*. *obtusa* essential fumigant (gray bars). *p<0.05, ***p<0.0001.

Effects of essential oil from *C*. *obtusa* on reproductive performance were examined since reproductive capacity is closely related to fitness in insects [26]. The average number of eggs laid per day by each female was not significantly affected by exposure to a low concentration of essential oil (Fig 4, 1–10 μg/ml). The average number of eggs laid per day by each female was 48.8 ± 8.62 for control flies but decreased to 40.38 ± 5.03 (ANOVA test, p < 0.05) and 9.88 ± 2.78 (ANOVA test, p <0.0001) upon exposure to fumigants of essential oil at concentrations of 25 or 70 μg/ml, respectively (Fig 4). Furthermore, flies exposed to 25 or 70 μg/ml of *C*. *obtusa* essential oil fumigant died within 4–5 or 1–2 days, respectively.

### Effects of *C*. *obtusa* essential oil on avoidance behavior

**Fig 5:**
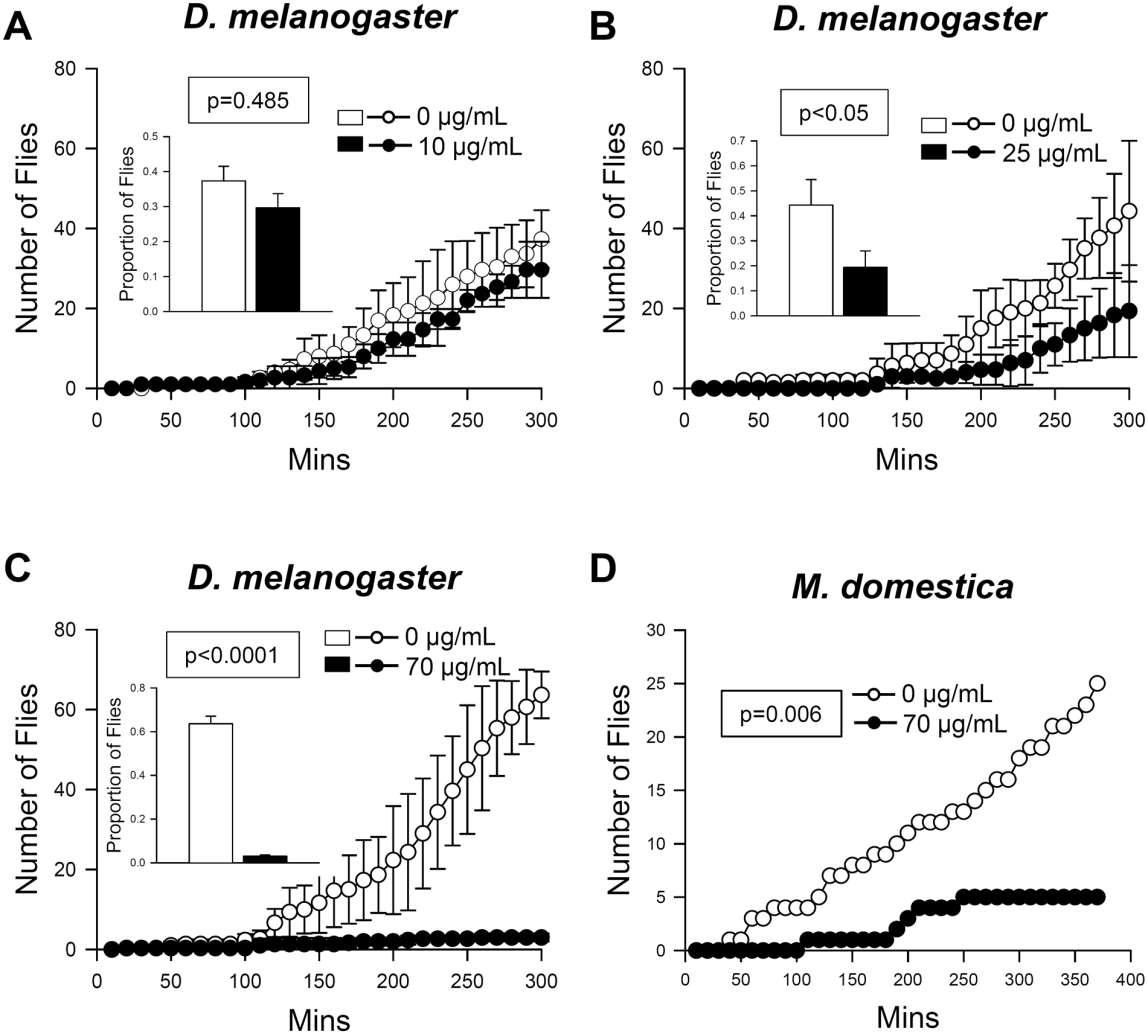
Essential oil from *C*. *obtusa* repels fruit flies and house flies. Fumigant of *C*. *obtusa* essential oil caused avoidance behaviors in fruit flies (A-C, *D*. *melanogaster*) and house flies (D, *M*. *domestica*). Inset graphs show the proportion of flies on each side at 300 minutes after setup. P values from a Chi-squared test are presented for each test.

The repellent activity of essential oil from *C*. *obtusa* was examined. Based on T-maze assay, a choice chamber was designed to give flies two irreversible choices, one with essential oil and one without. The numbers of flies choosing each diet containing vial were recorded for 5 hours every 10 min. While fumigant of 10 μg/ml essential oil did not affect the avoidance behavior of fruit flies, fruit flies significantly avoided essential oil fumigant at 25–70 μg/ml in a dose-dependent manner (Fig 5A–5C). In addition, similar avoidance behavior was observed when house flies, *M*. *domestica*, were given a choice (Fig 5D). These results indicate that fumigant of *C*. *obtusa* essential oil has powerful repellent activity against flies.

### Duration time of essential oil fumigants

**Fig 6:**
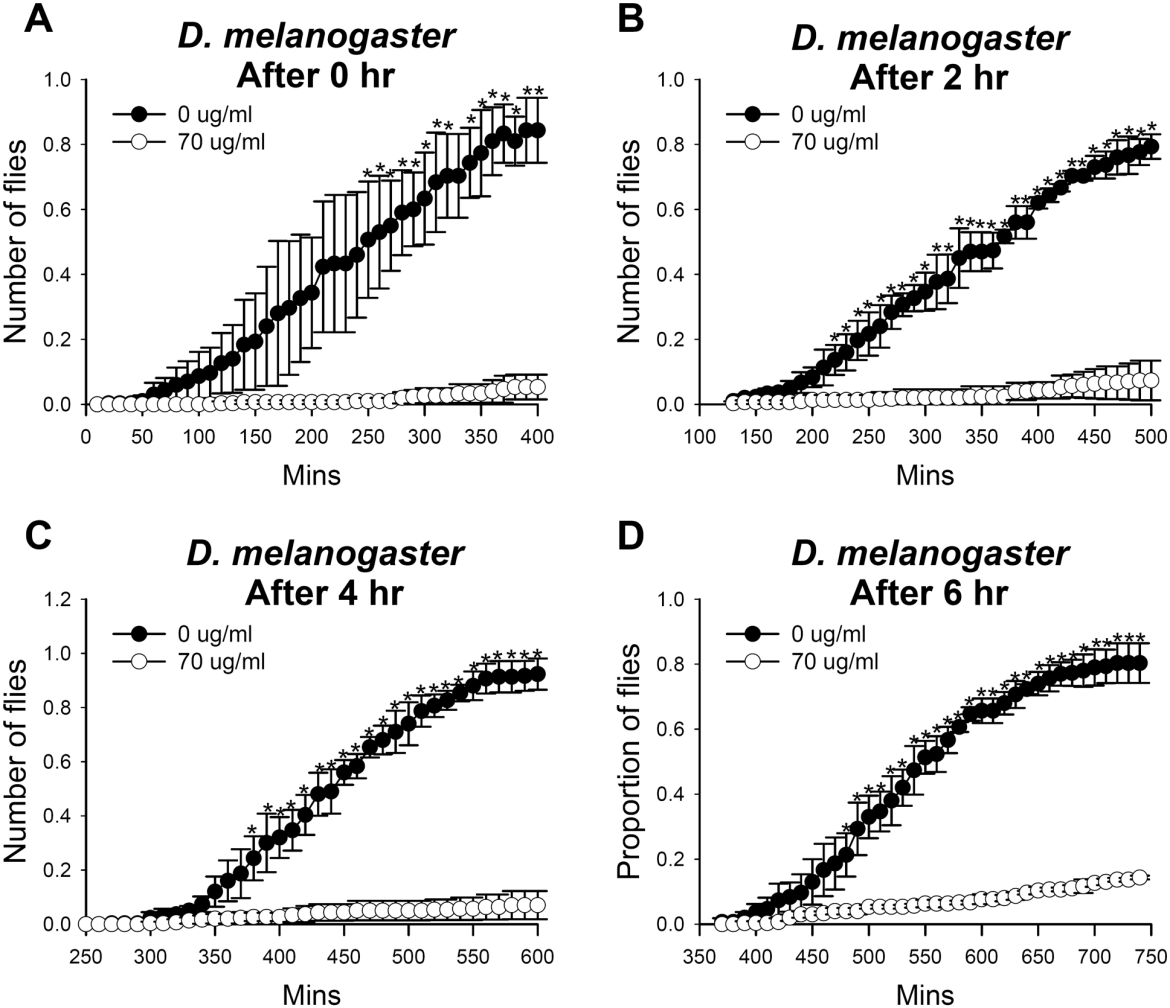
Repellent activity of *C*. *obtusa* essential oil lasts for 5 hours. Number of *D*. *melanogaster* choosing *C*. *obtusa* essential oil-containing vial was counted immediately or 2, 4, or 6 hours after administration of essential oil. Almost no flies were observed in *C*. *obtusa* essential oil-containing vial for 5 hours.

Use of essential oils as insect repellents has an efficacy problem since most essential oils are highly volatile [27]. We therefore examined the duration of repellency of *C*. *obtusa* essential oil fumigants using the choice chamber equipped with a funnel, similar to the experiment for repellent activity. Fruit flies were transferred to a choice chamber and given the choice immediately or 2, 4, or 6 hours after administration of 70 μg/ml *C*. *obtusa* essential oil. The flies started to enter the essential oil-containing vial at 280–300 min after essential oil administration (Fig 6A). Similarly, repellent activity was initially reduced (i. e. flies started to choose essential oil-containing vial) at ~ 300 min after essential oil administration (Fig 6B–6D). This indicates that fumigants of *C*. *obtusa* essential oil maintained avoidance activity for at least 5 hours.

## Discussion

Hinoki cypress (*Chamaecyparis obtusa*) is a familiar tree to the public due to its popularity in forest bathing in East Asia countries, including Korea, Japan, and China. Essential oils from other *Chamaecyparis* species have been reported to have insecticidal and antimicrobial activities. For example, essential oil from *Chamaecyparis lawsoniana* has larvicidal and repellent activities against Asian tiger mosquito, *Aedes albopictus* [28], as well as antibacterial activities against *Bacillus subtilis*, *Staphylococcus aureus*, and *Micrococcus luteus* [29]. Essential oil from *Chamaecypris formosensis* also has growth inhibitory activity against phytopathogenic fungi [30]. In this study, we showed that ingestion and fumigation of essential oil from *Chamaecyparis obtusa* has insecticidal activity in fruit flies, *D*. *melanogaster*. Similarly, Park and colleagues demonstrated the insecticidal activity of *C*. *obtusa* essential oil against two pests, *Callosobruchus chinensis* and *Sitophilus oryzae* [17]. They showed that exposure to *C*. *obtusa* essential oil at a dose of 38 mg/ml resulted in mortality rates of 29% in *Callosobruchus chinensis* and 6% in *Sitophilus oryzae*. Intriguingly, it was recently reported that hot water extract of *C*. *obtusa* has antioxidant activity and extends the lifespan of *C*. *elegans* [31]. Several phytochemicals (catechin, quercetin, and myricetin) in the water extract may be responsible for the antioxidant and life-extending effects of *C*. *obtusa* in *C*. *elegans*, but phytochemicals were not found in essential oil used in our study (Table 1).

Fumigation of *C*. *obtusa* essential oil did not significantly reduce development of *Drosophila* larvae (Fig 2A). This can be attributed to limited access to the odor, since larvae reside within semi-solid food during most of their lives. Therefore, we investigated the effect of parental odor exposure on development of offspring. Intriguingly, the developmental ratio of eggs to pupae was significantly reduced in a dose-dependent manner upon parental exposure to essential oil fumigant (Fig 2C). Several reports have studied the effects of essential oil on development of insects [27], but our findings are the first on the transgenerational effect of essential oil.

Fumigation of *C*. *obtusa* essential oil reduced locomotive activities of both sexes of fruit flies (Fig 3). Likewise, the neurotoxic effect of essential oil on insects has been documented in several studies. For example, *Lippia turbinate* essential oil was shown to reduce ambulation speed and total time in mosquito larvae (Kembro et al., 2009). In contrast to its neurotoxic effect on insects, essential oil from *C*. *obtusa* has been reported to have beneficial effects on neuronal health of mammals. Inhalation of *C*. *obtusa* essential oil can reduce anxiety-related behaviors [32], Alzheimer-related neuronal cell apoptosis, and memory dysfunction in rats [33]. In addition, biflavonoids from *C*. *obtusa* leaves were reported to have neuroprotective activity against glutamate-induced oxidative stress in mouse hippocampal cells [34], and essential oil from *C*. *obtusa* has anxiolytic-like and stress mitigation effects in mice [35]. These findings suggest that the underlying mechanisms of *C*. *obtusa* on neuronal function seem to be different between mammals and insects.

Fumigation of *C*. *obtusa* essential oil showed strong repellent effects on *D*. *melanogaster* and *M*. *domestica* (Fig 5). These results indicate that essential oil from *C*. *obtusa* can be used as a natural fly repellent. Many essential oils may not be suitable to be used as repellents due to their volatile nature and short half-life. However, essential oil from *C*. *obtusa* provided 100% repellency for 5 hours against *D*. *melanogaster* (Fig 6), comparable to the duration time of N,N,-ditethyl-meta-toluamide (DEET) [36]. Likewise, *Cymbopogen* plants provided repellent activity for 2–12 hours against mosquitoes [37].

Compositions of essential oils can differ by harvesting season, geographical source, and sampling part of the plant [5, 38, 39]. By GC-MS, the main components of *C*. *obtusa* essential oil used in this study were identified as α-Terpinolene (19.45%), (+)-3-Carene (15.17%), α-Pinene (10.12%), Sabinene (6.27%), and γ-Terpinene (4.77%) (Table 1). Similarly, several studies have showed that the major components of *C*. *obtusa* essential oil in Korea are Sabinene and (+)-2-Carene [40], α-Terpinyl acetate and Sabinene [33, 41, 42], (+)-2-Carene and Sabinene [40], and Limonene, Bornyl acetate, and Sabinene [17]. Since bornyl acetate, Terpinolene, and α-Phellandrene induce high mortality in *C*. *chinensis* and *S*. *oryzae* while Sabinene, α-Pinene, and Myrcene do not [40], it will be interesting to determine the active components responsible for the insecticidal and repellent activities of *C*. *obtusa* essential oil.

Taken together, our data show that essential oil from *C*. *obtusa* has insecticidal activity and affects the fecundity, locomotive behavior, and development of fruit flies. In addition, essential oil has strong repellent activity in fruit flies and house flies with a duration time up to 5 hours. Together with the possible beneficial effects of *C*. *obtusa* on human health, our results suggest that *C*. *obtusa* essential oil can be potentially used as a ‘human-friendly’ insect repellent.
